# Draft genome sequence of thermotolerant *Saccharomyces cerevisiae* AH465, isolated from Mt. Tatsuda, Kumamoto, Japan

**DOI:** 10.1128/mra.01124-23

**Published:** 2024-03-19

**Authors:** Yu Sasano, Ayuki Hamaguchi, Hisataka Taguchi

**Affiliations:** 1 Department of Biotechnology and Life Sciences, Faculty of Biotechnology and Life Sciences, Sojo University, Kumamoto, Japan; University of Maryland School of Medicine, Baltimore, Maryland, USA

**Keywords:** yeast, thermotolerance, hybrid assembly

## Abstract

Thermotolerance is a required characteristic for biofuel production by yeast. Here, we report the draft genome sequence of thermotolerant *Saccharomyces cerevisiae* AH465, which was originally isolated by the authors. A hybrid assembly approach using MinION Mk1b and MiSeq sequencers was conducted. The assembled sequence comprises 13.4 Mb in 26 contigs.

## ANNOUNCEMENT

*Saccharomyces cerevisiae* is one of the most important microbes as a host for producing useful materials, such as biofuel. Owing to the difference in optimal temperatures between saccharification and fermentation, a thermotolerant yeast is desirable for biofuel production (1, 2). We isolated *Saccharomyces cerevisiae* AH465 from black and muddy water pooled in the hollow of a rotting tree in Mt. Tatsuda, Kumamoto, Japan. The aliquot of the pooled water was streaked onto YMA agar plates (3), supplemented with 100 µg/mL ampicillin and 50 µg/mL chloramphenicol to inhibit the growth of bacteria, and 200 µg/mL sodium propionate to inhibit that of filamentous fungi, cultivated for several days, and finally, single colony was isolated. Sequencing analysis to determine rDNA sequence using primer pairs ITS5 (4) and LR5 (5) revealed that AH465 is *Saccharomyces cerevisiae*. Sequence of AH465 showed 100% identity for D1/D2 region and 99.9% identity (only one substitution) for ITS region as compared to that of many *S. cerevisiae* strains. AH465 is able to grow vigorously above 42°C under aerobic condition and at 41°C under anaerobic condition and produce ethanol of approximately 90% of the theoretical yield under these conditions. These characteristics of AH465 are useful for biofuel production; therefore, genetic mechanism underlying this remarkable thermotolerance is to be elucidated.

The genomic DNA of AH465 was extracted from cells grown overnight on Yeast extract-peptone-dextrose (YPD) liquid medium at 30°C by Dr. GenTLE (TakaraBio). The DNA library for Oxford Nanopore Technologies sequencing was prepared using Ligation Sequencing Kit (SQK-LSK109). Long-read sequencing was performed using a MinION Mk1b sequencer on a R9.4 flow cell (FLO-MIN106). Basecalling was performed by Guppy v6.5.7 (6) with hac mode, yielding 32,888 reads with an N_50_ of 34,466 bp (coverage, 32×). Hereafter, default parameters were used except where otherwise noted when using a software. Adaptor sequences were trimmed by Porechop (v0.2.4; https://github.com/rrwick/Porechop), and trimmed reads were assembled with Flye v2.9.2 (7). The DNA library for Illumina sequencing was prepared using Nextera XT DNA Library Prep Kit (FC-131-1024). Short-read sequencing was performed using Illumina MiSeq system with MiSeq Reagent Kit v3 (150 Cycles) (MS-102-3001), yielding 6,428,011 read pairs (paired-end [PE], 2 × 75 bp format; coverage, 80×). For both long- and short reads, the read quality was checked using FastQC (v0.12.1; https://github.com/s-andrews/FastQC). The assembled sequence obtained by Flye was polished with the short reads four times using Pilon v1.24 (8). The hybrid assembly resulted in 26 contigs with a total length of 13.4 Mb (N_50_, 790,683 bp; G + C content, 38.07%; coverage, 112×). Analysis of the genome completeness evaluated by BUSCO v5.4.6 (9) with the saccharomycetes_odb10 data set resulted in a value of 99.2%. We analyzed structural variations (>1 kb) by Sniffles v2.2 (10) by comparison with YPS128 strain, which is one of the most similar to AH465, genome-sequenced and non-thermotolerant strain (11). We found 13 large insertions and 30 large deletions. The genomic information of *S. cerevisiae* AH465 will give a useful insight into the elucidation of thermotolerant mechanism in yeast and lead to molecular breeding of yeast strains for industrial biofuel production.

## Data Availability

This whole-genome sequencing project has been deposited in DDBJ/ENA/GenBank under BioProject accession number PRJDB17000, and BioSample accession number SAMD00657001. The raw sequencing data are available under the SRA accession numbers DRX496868 and DRX496869. The draft genome sequences of Saccharomyces cerevisiae AH465 strain has been deposited in DDBJ/ENA/GenBank under accession numbers BTXB01000001-BTXB01000026.
